# Pilot testing of the Becoming Breastfeeding Friendly toolbox in Ghana

**DOI:** 10.1186/s13006-018-0172-y

**Published:** 2018-07-11

**Authors:** Richmond Aryeetey, Amber Hromi-Fiedler, Seth Adu-Afarwuah, Esi Amoaful, Gifty Ampah, Marian Gatiba, Akosua Kwakye, Gloria Otoo, Gyikua Plange-Rhule, Isabella Sagoe-Moses, Lilian Selenje, Rafael Pérez-Escamilla, Ghana Becoming Breastfeeding Friendly Committee

**Affiliations:** 1 School of Public Health, University of Ghana, Legon, Accra, Ghana; 2 Yale University School of Public Health, New Haven, CT USA; 3 Nutrition and Food Science Department, University of Ghana, Legon, Accra, Ghana; 4 Nutrition Department, Ghana Health Service, Accra, Ghana; 5 Food and Drugs Authority, Accra, Ghana; 6 World Health Organization, Accra, Ghana; 7 School of Medical Sciences, Kwame Nkrumah University of Science and Technology, Kumasi, Ghana; 8 Reproductive and Child Health Department, Ghana Health Service, Accra, Ghana; 9 United Nations Children’s Fund, Accra, Ghana

**Keywords:** Scale-up, Breastfeeding, Bench marking, Policy, Evidence, Ghana

## Abstract

**Background:**

Ghana has achieved significant progress in breastfeeding practices in the past two decades. Further progress is, however, limited by insufficient government funding and declining donor support for breastfeeding programs. The current study pretested feasibility of the *Becoming Breastfeeding Friendly (BBF) toolbox* in Ghana, to assess the existing enabling environment and gaps for scaling-up effective actions.

**Methods:**

Between June 2016 and April 2017, a 15-person expert country committee drawn from government and non-government agencies was established to implement the BBF toolbox. The committee used the BBF index (BBFI), comprising of 54 benchmarks and eight gears of the Breastfeeding Gear Model (advocacy; political will; legislation and policy; funding and resources; training and program delivery; promotion; research and evaluation; and coordination, goals and monitoring). Available evidence (document reviews, and key informant interviews) was used to arrive at consensus-scoring of benchmarks. Benchmark scores ranged between 0 (no progress) and 3 (major progress). Scores for each gear were averaged to estimate the Gear Total Score (GTS), ranging from 0 (least) to 3.0 (strong). GTS’s were aggregated as a weighted average to estimate the BBFI which ranged from 0 (weak) to 3.0 (outstanding). Gaps in policy and program implementation and recommendations were proposed for decision-making.

**Results:**

The BBFI score was 2.0, indicating a moderate scaling-up environment for breastfeeding in Ghana. Four gears recorded strong gear strength: advocacy (2.3); political will (2.3); legislation and policy (2.3); and coordination, goals and monitoring (2.7). The remaining four gears had moderate gear strength: funding and resources (1.3); training and program delivery (1.9); promotion (2.0); and research and evaluation (1.3). Key policy and program gaps identified by the committee included sub-optimal coordination across partners, inadequate coverage and quality of services, insufficient government funding, sub-optimal enforcement of policies, and inadequate monitoring of existing initiatives. Prioritized recommendations from the process were: 1) strengthen advocacy and empower breastfeeding champions, 2) strengthen breastfeeding regulations, including maternity protection, 3) strengthen capacity for providing breastfeeding services, and 4) expand and sustain breastfeeding awareness initiatives.

**Conclusions:**

The moderate environment for scaling-up breastfeeding in Ghana can be further strengthened by addressing identified gaps in policy and programs.

**Electronic supplementary material:**

The online version of this article (10.1186/s13006-018-0172-y) contains supplementary material, which is available to authorized users.

## Background

There already exists strong evidence linking optimal child health and development outcomes with ideal breastfeeding behaviors such as early initiation at birth, exclusive breastfeeding during the first six months of life, and breastfeeding for at least two years [1, 2]. The benefits of improved breastfeeding practices extends beyond the breastfed child, both in the short and long term, to their mothers, and the society at large as indicated by reduced morbidity and mortality, improved quality of life, and enhanced human capital [2–4]. The 2016 *Lancet* series on breastfeeding provides evidence that children who are breastfed appropriately for a longer duration have a lower risk of obesity and diabetes later in adult life, have lower risk of dental malocclusion, and have higher intelligence (children and adolescents) than those who are breastfed for shorter duration [1, 5]. The same review also linked breastfeeding with positive maternal outcomes including lower risk of breast and ovarian cancer, diabetes, and increased birth spacing. These benefits justify the World Health Organization’s (WHO) strong recommendation to implement programs which protect, promote and support optimal breastfeeding practices [6]. In low-income countries where child survival and development are often threatened by chronic exposure to infectious diseases and inadequate diets, the benefits of improved breastfeeding practices are even greater [7–9]. Increasing optimal breastfeeding behaviors globally through effective scaling-up frameworks could save up to 823,000 children under five years of age from preventable deaths as well as avert about 20,000 breast cancer deaths, annually [1]. Therefore, promoting, protecting, and supporting breastfeeding has the potential to save countless lives, and positively affect a country’s overall population well-being and development.

Current estimates, however, show that the prevalence of optimal breastfeeding practices is low across world regions. Early initiation of breastfeeding is lowest in lower middle-income countries (< 40%). Only 37% of children under six months of age in low- and middle-income countries are breastfed exclusively [4]. Furthermore, more than 40% of children between 20 and 23 months of age worldwide do not receive the benefits of continued breastfeeding [10]. In addition, breastfeeding practice is increasingly threatened by the promotion of breast milk substitutes [2]. Globally, implementation of programs that strengthen the breastfeeding environment remains suboptimal. For example, only 53% of countries world-wide meet the International Labor Organization (ILO) minimum standard of 14 weeks paid maternity leave [11]. Thus, many working women do not have adequate maternity protection to breastfeed their infants as recommended. Despite being endorsed globally over 35 years ago, only 39 out of 194 countries have comprehensive legislation to protect against unethical marketing of breast milk substitutes [12]. Thus, nursing mothers are exposed to unethical marketing of breast milk substitutes that violate the WHO Code [13].

Currently, effective strategies exist to achieve optimal breastfeeding practices, which include: 1) counseling, support and management services targeting individual mothers, 2) legislative, policy, and financing programs which address barriers at community and institutional levels, and 3) social mobilization and mass media strategies which address sociocultural and market-driven barriers to optimal breastfeeding [2]. If integrated, scaled-up, and monitored, the appropriate combination of strategies can strengthen the national environment to promote, protect, and support optimal breastfeeding behavior [14].

Becoming Breastfeeding Friendly (BBF) is a guide to assist countries in assessing their readiness and tracking their progress in scaling-up their national breastfeeding protection, promotion, and support programs, policies, and initiatives [15]. The BBF is grounded in the evidence-based Breastfeeding Gear Model (BFGM) framework which posits that eight gears must work together in harmony for a country to fully scale-up their breastfeeding programs [14]. The BBF provides countries with a toolbox that consists of an index, case studies, and a 5-meeting process to guide them in the step-by-step application of the BBF toolbox. In 2016, the BBF toolbox was pilot tested in Ghana as a partnership between University of Ghana, Yale University, and the Ghana Health Service (GHS) and its partner United Nations and Donor agencies. Ghana was an appropriate setting to pilot test the BBF toolbox given the recent decline in exclusive breastfeeding rates to 52% [16] after decades of successful and steady increases where rates reached 64% in 2008 [17]. Since the early 1990s, Ghana strengthened breastfeeding policy and services in full commitment to improving breastfeeding practices, nation-wide. However, with recent breastfeeding rates declining [16, 17], BBF provided Ghana with a guide to identify gaps and propose policy recommendations that are needed to strengthen the breastfeeding environment. This paper describes the implementation process and outcomes of the BBF toolbox testing in Ghana.

## Methods

### Study site

The Republic of Ghana is located on the Western Coast of Africa. Based on the 2010 population and housing census, the current population is estimated at about 26 million people [18]. The world bank classifies Ghana as a lower middle-income country [19]. Ghana’s health system is considered to be reasonably well developed when compared with other countries in Sub-Saharan Africa [20]. However, its performance is below that of other countries of similar income and health expenditure outside Sub-Saharan Africa. Recent evidence shows mixed performance regarding health outcomes in Ghana. While life expectancy has been gradually increasing (60 years for males; 63 years for females), there is a persistently high rate of deaths among women and children [16]. Most recent data estimates a maternal mortality rate of 164/100,000 live births and under-five child mortality of 60/1000 live births. The health system in Ghana is designed to deliver preventive health services through decentralized district and municipal service centers with the aim to increase access to services. A core component of preventive maternal and child health services is communication and support for breastfeeding. Breastfeeding services are decentralized across multiple government agencies with support from non-government partners across sectors and administrative levels.

The Ministry of Health (MoH) and the GHS are the leading agencies for developing policies, legislation, and strategies for breastfeeding in Ghana [20]. Other agencies (both government and non-government) support the MoH and GHS in developing and enforcing breastfeeding policies, as well as providing technical and financial assistance for breastfeeding. Breastfeeding promotion and communication, as well as other support services, in communities and facilities, are provided, mainly, by GHS facilities as well as Teaching Hospitals which are managed by the MoH. Outside of the health sector, the Ministry of Gender, Children, and Social Protection (MGCSP) together with the Ministry of Employment and Labour Relations (MELR) works collaboratively with the MoH to promote maternity protection for breastfeeding in the workplace. The Food and Drugs Authority (FDA) also plays a regulatory role of enforcing compliance to the National Breastfeeding Regulation 2000 (LI 1667) [21] and the International Code of Marketing of Breast Milk Substitutes [13]. The activities of these government agencies are complemented by technical and financial support from multiple development partners (United Nations Agencies, Bilateral donors, and local and international Non-Governmental Organizations).

### BBF implementation in Ghana

The process for implementing the BBF toolbox is as outlined in the BBF implementation manual [22]. The BBF toolbox is based on the evidence-based BFGM which was developed through a rigorous consultative process involving international experts across diverse areas associated with lactation [15]. The BFGM stipulates that eight ‘*gears’* must work harmoniously to achieve a country-level scale-up of breastfeeding. The BBF toolbox is designed to estimate the BBF index (BBFI) which is an aggregate score based on 54 specific benchmarks: advocacy (4 benchmarks); political will (3 benchmarks); legislation and policy (10 benchmarks); funding and resources (3 benchmarks); training and program delivery (17 benchmarks); promotion (3 benchmarks); research and evaluation (10 benchmarks); and coordination, goals and monitoring (3 benchmarks). Based on the existing situation, each benchmark is scored as 0 (no progress); 1(partial progress), 2 (minimal progress), and 3 (major progress). Benchmark scores for each gear are then averaged to estimate the Gear Total Score (GTS): 0 (gear not present), 0.1 to 1.0 (weak gear strength), 1.1 to 2.0 (moderate gear strength), and 2.1–3.0 (strong gear strength). The GTS’s are then aggregated as a weighted average to estimate the total BBF score which ranges from 0 to 1.0 (weak scaling-up environment) 1.1–2.0 (moderate scaling-up environment), 2.1–2.9 (strong scaling-up environment, to 3.0 (outstanding scaling-up environment).

A key component of BBF implementation is the country committee which, in Ghana, comprised of experts from nine agencies involved in breastfeeding programming in both government and non-government agencies. The composition of the country committee is indicated in Table 1. The committee utilized available documents, expert opinion, and case studies of best practices to arrive at their decisions on the status of different aspects of the breastfeeding scale-up environment. The committee achieved this through participation in five scheduled meetings over a period of 11 months to generate GTSs for the BBFI, identify gaps in breastfeeding programming in Ghana, and develop and prioritize policy and program recommendations to address the identified gaps [15]. In between these scheduled meetings, four teams, each with a membership of three committee members, collected data (evidence from document review, and key informant interviews), and had meetings to work on scoring assigned gears and its constituent benchmarks. Recommendations were presented to key high-level stakeholders to guide strategy development and prioritization of actions to ensure a breastfeeding friendly environment.

**Table 1:** Becoming breastfeeding friendly committee membership and meeting participation

Institution	Number of staff	Committee meetings attended					
		First	Second	Third	Fourth	Fifth	High level meeting
UNICEF^a^	2	1	1	0	1	1	0
		0	1	1	0	1	0
University of Ghana	2	1	1	1	1	1	0
		1	1	1	1	1	0
WHO^b^	1	1	1	0	1	1	1
Ghana Health Service	3	1	1	0	1	0	1
		1	1	0	1	1	0
		1	1	0	1	1	1
USAID^c^	1	1	1	0	1	1	1
Food and Drugs Authority	2	1	0	0	0	0	0
		0	0	1	1	1	0
Korle-bu Teaching Hospital	2	1	1	1	1	1	0
		0	0	1	0	0	0
Komfo Anokye Teaching Hospital	1	1	1	1	1	1	0
World Food Program	1	0	0	1	0	0	0

^a^UNICEF=United Nations Children’s Fund

^b^World Health Organization

^c^United States Agency for International Development

### Committee meetings and gear scoring process

The BBF 5-meeting process was conducted in Ghana between June 2016 and January 2017. Preparatory activities, led by the in-country investigator, occurred earlier in March to June 2016 and involved identifying, and sensitizing key stakeholders, and inviting them to participate as country committee members. Following this, an initial list of 12 key stakeholder institutions was generated. Thereafter, each of the stakeholders/stakeholder institutions was consulted individually and provided with a brief overview of BBF and to confirm their willingness to participate. During the process, some participants who were unable to attend some of the committee meetings either sent notice to be absent or were represented by another person from their institution. As a result, by the end of the process, 15 persons from 9 institutions participated in the process (Table 1).

**Fig. 1:**
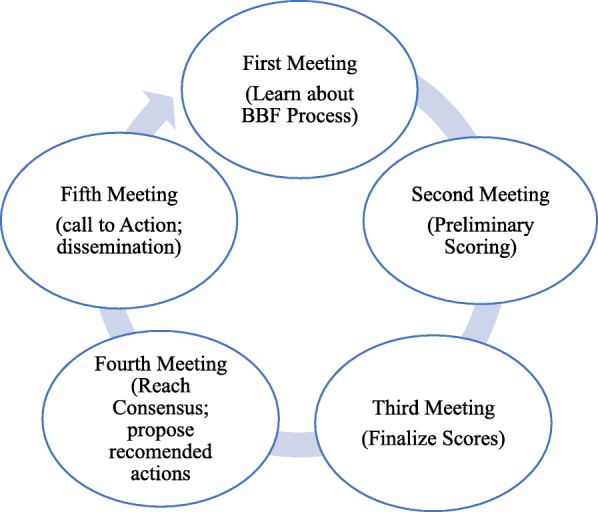
The Becoming Breastfeeding Friendly Process implemented in Ghana

The five-meeting process (Fig. 1) was designed to help countries reach consensus on benchmark and gear scoring over the course of 11 months, starting in June 2016 with the first country committee meeting. The aim of this 2-day meeting was four-fold. First, it was to build the capacity of the committee on the BFGM, the gears and benchmarks of the BBFI, as well as the process for scoring the benchmarks. Secondly, the meeting defined and discussed the roles and expectations of country committee members. Thirdly, during this meeting, country committee members were assigned to their respective gear teams which comprised of three members per team, with each team being allocated to work on at least 1 gear (minimum of 1 and maximum of 3 gears per team). Finally, the meeting provided opportunity for committee members to develop data gathering action workplans in which the teams identified potential data sources, data collection strategies, a timeline to review collected data and reach consensus on preliminary scores for each benchmark within their assigned gears. A gear team leader was nominated to coordinate each team’s activities, provide leadership in identifying the evidence needs of the team, communicate team progress with the Ghana BBF coordination team, document proceedings of team meetings, and present output of team work at subsequent country committee meetings. Following the workplan development, gear teams shared their workplans with the entire country committee for discussion. Input was received from other committee members as well as the Ghana BBF coordination team prior to its finalization.

Two months later, the second meeting was convened in August 2016, for gear teams to present their data gathering progress and preliminary benchmark scores (Table 2). While most teams had made significant progress with scoring their assigned benchmarks by this time, one team (training and program delivery gear) had scored less than 20% of the gear benchmarks due to limited data available for the scoring process. Scoring progress and data gathering strategies were discussed to ensure teams had access to additional data needed to score remaining benchmarks. When consensus could not be reached on specific benchmark scores, the country committee discussed additional data source options and teams were encouraged to consult these additional data sources as they work further on completing their benchmark scoring.

**Table 2:** Becoming Breastfeeding Friendly Committee team progress in completing tasks

Teams	Gears assigned	Committee Meetings				
		First	Second	Third	Fourth	Between Fourth and Fifth
Team 1	Legislation and policy (10 benchmarks)	All teams attended training, developed team work plan and submitted to BBF^1^ Coordination	90% (*n* = 9) benchmarks scored; insufficient data to score 1 benchmark	90% (*n* = 9) of benchmarks scored. Additional data sources recommended for scoring	Consensus reached by BBF^a^ committee on 90% (*n* = 9) of benchmarks	Consensus reached by committee on 100% (*n* = 10) of benchmarks
	Funding and resources (4 benchmarks)		100% (*n* = 4) benchmarks scored	100% (*n* = 4) of benchmarks scored. Additional data sources recommended to explain scores	Consensus reached by BBF committee on 100% (*n* = 4) of benchmarks	–
Team 2	Advocacy (4 benchmarks)		100% (*n* = 4) of benchmarks scored	100% (*n* = 4) of benchmarks scored; Data gaps related to scores reported	Consensus reached by BBF committee on 100% (*n* = 4) of benchmarks	–
	Political will (3 benchmarks)		100% of benchmarks scored	100% (*n* = 3) of benchmarks scored; Data gaps related to scores reported	Consensus reached by BBF committee on 100% (*n* = 3) of benchmarks.	–
	Promotion (3 benchmarks)		100% of benchmarks scored	100% (*n* = 3) of benchmarks scored. Data gaps related to scores reported	Consensus reached by BBF committee on 100% (*n* = 3) of benchmarks	–
Team 3	Training and program delivery (17 benchmarks)		18% (3) of benchmarks scored; insufficient data to score 14 benchmarks	76% (*n* = 13) of bench marks scored	Consensus reached by BBF committee on 47% (*n* = 8) of benchmarks	Consensus reached by BBF committee on 100% (*n* = 17) of benchmarks
Team 4	Research and evaluation (10 benchmarks)		80% (*n* = 8) of benchmarks scored; insufficient data to score 2 benchmarks	90% (*n* = 9) of benchmarks scored; questions raised about robustness of data used for scoring	Consensus reached by BBF committee on 50% (*n* = 5) of benchmarks	Consensus reached by BBF committee on 100% (*n* = 10) of benchmarks
	Coordination, goals and monitoring (3 benchmarks)		None of the benchmarks scored	100% (*n* = 3) of benchmarks scored	Consensus reached by BBF committee on 66% (*n* = 2) of benchmarks	Consensus reached by BBF committee on 100% (*n* = 3) of benchmarks

^a^BBF=Becoming Breastfeeding Friendly

One month later, in September 2016, the committee met for the third time for a one-day meeting where teams presented progress on benchmark scores. Prior to the team presentations, the BBF toolbox case studies were presented and provided to country committee members as a tool to assist teams in finalizing their benchmark scores. At the end of presentations, seven benchmarks still lacked accessible data needed to complete the scoring. Alternate data collection source options were discussed, and teams were tasked with: a) finalizing benchmark scores where consensus had not been reached and b) developing key gaps and recommendations for their benchmarks and gears for presentation at the 4th meeting.

The country committee convened for their fourth meeting one month later in November 2016. At this one-day meeting, each benchmark was discussed thoroughly, and final consensus was reached on scores for 38 of the 54 benchmarks. For the 16 benchmarks where consensus was not reached, adequate data had not been found yet to either support scoring these benchmarks or to clearly define the gaps related to the benchmark. The committee agreed for the Ghana BBF coordination team to work directly with the respective gear teams, after the fourth meeting, to provide the evidence needed to arrive at the proposed scores. Following this process, the two gear teams secured the needed data and the scores, and data gaps details on the remaining 16 benchmarks were confirmed through email communication with the country coordination team. Since it was not possible to discuss the gaps and recommendations at the 4th meeting due to time constraints, gear teams independently developed and submitted recommendations for their respective gears to the BBF coordination team via email. A policy brief (Additional file 1: Appendix S1) and infographic (Additional file 2: Appendix S2 and Additional file 3: Appendix S3) describing the BBF GTSs and prioritized recommendations were developed in preparation for the 5th meeting.

Three months later, the country committee held their fifth meeting in February 2017. This was a call to action meeting in which key stakeholder institutions involved directly or indirectly with breastfeeding programming, as well as various media institutions were invited to receive and discuss the findings and recommendations of the BBF outcomes in Ghana. Participating stakeholders represented government, United Nations, civil society, and regulatory agencies, including MELR, the MGCSP, GHS, Trades Union Congress, United States Agency for International Development, Communicate for Health, and Ghana Infant Nutrition Action Network, WHO, United Nations Children’s Fund, International Labour Organization, FDA, and the Medical and Dental Council. Following the presentation of BBF methodology and findings, which included sharing GTSs, total country score, the rationale for the scores, the gaps identified, and prioritized recommended actions, stakeholders discussed potential strategies to address the identified gaps in the national breastfeeding program.

In April 2017, a meeting was organized to share the BBF process, findings, and prioritized recommendations with key high-level decision makers. The meeting was attended by the Minister for MGCSP, the Deputy Director General of the GHS, country representatives of ILO and WHO, convener of Ghana Editors Forum (a network of news media editors in Ghana), representative of Ghana Congress on Evangelisation Women’s Ministry (a women’s religious organization), and four of the 15 country committee members. Following this meeting, the Minister of MGCSP requested a concept note (which was developed by the Ghana BBF coordination team) to guide their Ministry in developing breastfeeding plans for children and women.

## Results

**Fig. 2:**
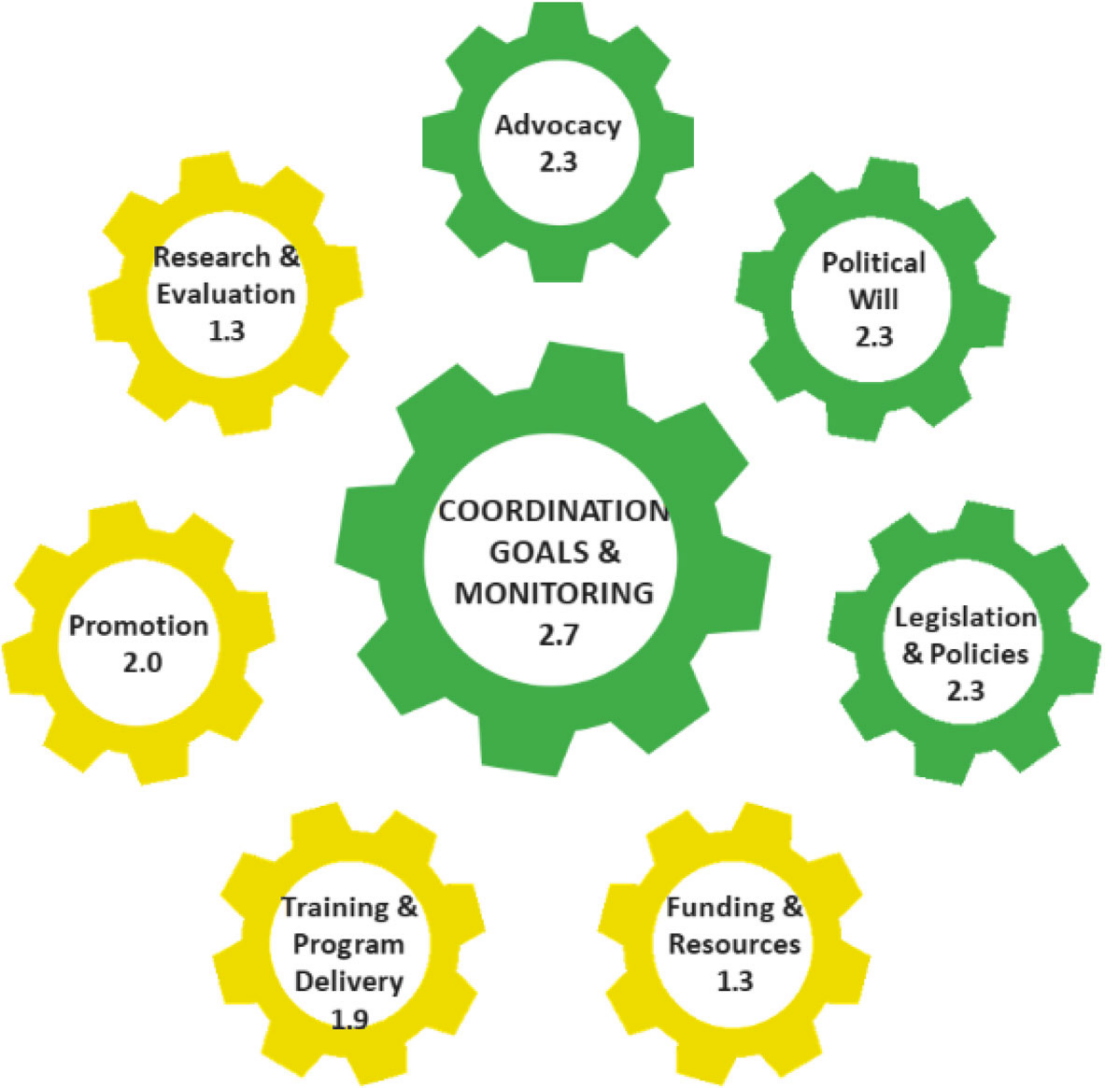
The Becoming Breastfeeding Friendly Gear Scores for Ghana, 2016. Gear Total scores: 0 = Gear not present; 0.1to 1.0 = Weak Gear Strength; 1.1 to 2.0 = Moderate Gear Strength; 2.1 to 3.0 = Strong Gear Strength

The BBFI score for Ghana was 2.0 indicating a moderate scaling- up environment for breastfeeding. Four of the gears received strong gear strength (> 2.1) and the remaining four had GTS’s classified as moderate (> 1.1 but < 2.0) (Fig. 2). The GTS’s showed that Ghana is strongest in coordination, goals, & monitoring, receiving a score of 2.7 out of 3.0. The funding & resources (score = 1.3) and research & evaluation (score = 1.3) gears scored the lowest, showing moderate gear strength. Gaps in breastfeeding program implementation were documented (Table 3). Across gears, there were initiatives that were already being implemented. However, the implementation of the identified initiatives was considered by the country committee as suboptimal with respect to their coordination, coverage, quality, funding, enforcement, and monitoring (Table 3).

**Table 3:** Gear strengths and gaps identified in the Becoming breastfeeding friendly pilot testing process in Ghana

Gear	Strengths	Gaps	Recommended actions
Advocacy	• Strong capacity for breastfeeding advocacy and advocates exists at highest levels of government	• There is no network of advocates and thus advocacy is not coordinated • Advocacy is not sustained • Advocacy mainly limited to world breastfeeding week celebration	• Engage and Build capacity of media practitioners • Promote breastfeeding through existing forums • Actively engage and train breastfeeding champions
Political will	• Political will is demonstrated by existing government initiatives • Key government staff are influencing breastfeeding policy development	• Actions by government staff has not translated into full action for breastfeeding	• Engage parliamentarians using policy briefs • Advocate for adoption of ILO convention on maternity protection (No.183)
Legislation and policy	• Strong policy and legislative environment identified (BFHI, the Code, maternity protection, etc) • Institutions exist to implement these policies/legislation	• Gaps identified in existing legislation with respect to current WHA resolutions • Duration of maternity leave is less than ILO minimum standard • Code not enforced nation-wide	• Revise LI 1667 to incorporate recent WHA resolutions • Revise penalties for LI 1667 violations • Strengthen implementation of the code • Facilitate adoption of at least 14 weeks maternity leave
Funding and resources	• At least one fully funded position for breastfeeding coordination and monitoring at national level	• No earmarked funding for breastfeeding at national or sub-national levels for government and private sector breastfeeding services	• Provide adequate funding for breastfeeding programs • Track expenditure on breastfeeding programming
Training and program Delivery	• Revised curricula for pre-service training in breastfeeding • In-service training activities has been implemented throughout the country • Breastfeeding is integrated into various existing programs at sub-national level • BFHI designation and implementation exists	• Revised curricula not being utilized in many training institutions • Coverage of in-service training remains sub-optimal and poorly tracked/coordinated • No clear definition of competence level of trainers • BFHI coverage is low and infrequently re-assessed	• Promote use of revised pre-service training curricula • Harmonize and Track coverage of breastfeeding capacity strengthening • Strengthen BFHI monitoring/re-assessment process
Promotion	• Several government initiatives (strategy documents) identified that aim to promote breastfeeding	• Identified initiatives are not adequately funded by government • Impact of these initiatives on awareness is sub-optimal	• Engage retired health staff to promote breastfeeding • Provide funding for promotion activities • Promote breastfeeding using maternity promotion platforms
Research and evaluation	• Indicators exists for regular (surveys), and routine (institutional data) monitoring of breastfeeding • BFHI/Ten Steps monitoring system exists	• Data exists for tracking progress in breastfeeding practice at national but not sub-national levels • No data on vulnerable groups • No tracking system for violations of maternity protection legislation • No tracking of BCC	• Implement planned annual breastfeeding surveillance system • Identify and track vulnerability to breastfeeding • Decentralize monitoring of the code • Track BCC activities
Coordination, goals, and monitoring	• Multi-sectoral BFHI Authority coordinates implementation of BFHI at national level; BFHI monitoring decentralized • IYCF task team provides guidance on breastfeeding policy at national level	• Committees met infrequently and on a need-to-act basis	• Ensure regular meetings of coordination bodies • Develop a workplan for action on breastfeeding

Four priority recommendations emerged from the country committee from a total list of 46 recommendations. One prioritized recommendation focused on the need to enlist and build capacity of breastfeeding champions to enhance breastfeeding advocacy as well as increase promotion of breastfeeding as an effective child feeding strategy. The committee suggested that strategies for this recommendation include: a) increased partnership with journalists and the media, b) active promotion of breastfeeding through health professional society meetings, c) the use of social media as a key channel to expand breastfeeding promotion efforts. The second priority recommendation was to strengthen breastfeeding regulations. This requires ratifying the ILO maternity protection convention 183, increasing maternity leave beyond the current 12 weeks provided by the National Labour Law (ACT 658) and strengthening enforcement and monitoring of the Law. A key aspect of the maternity protection recommendation was to strengthen the baby-friendliness of workplaces. A handful of institutions which had dedicated breastfeeding rooms for working mothers were identified and were cited as examples which could be promoted as early adopters to encourage other workplaces, particularly, government agencies to strengthen the baby-friendliness of workplaces. The third priority recommendation emphasized strengthening capacity for delivering breastfeeding services. Utilization of revised pre-service training curricula, harmonization of in-service training curricula and training tools, coordination of in-service training initiatives, and certification of lactation consultants were identified as key strategies to enhance capacity in breastfeeding promotion and service delivery. Fourth and finally, the committee also prioritized scaling-up breastfeeding promotion activities using diverse channels.

## Discussion

BBF was pretested in Ghana to determine its feasibility and to generate evidence for action regarding breastfeeding scale-up in Ghana. The findings demonstrate the capacity of BBF to engender a collaborative cross-sectoral process for examining breastfeeding program components. The BBF process, generated a widely accepted measure of breastfeeding program implementation status in Ghana among stakeholders. In addition, the process yielded results describing the breastfeeding environment across various gears. This allows decision-makers to target resources and actions related to the identified gaps. Overall, the process demonstrated that Ghana has a moderately strong scale- up environment for breastfeeding. Participants at the fifth BBF meeting validated the findings as an accurate description of the breastfeeding program implementation situation in Ghana.

Further, several implementation gaps were identified as being related to the various gears. Regarding advocacy and promotion gears, it was identified that there is insufficient social mobilization regarding breastfeeding. Although there are influential individuals who have spoken about breastfeeding, there are no highly visible champions for breastfeeding. This lack of sufficient advocacy for breastfeeding may also explain why there is limited promotion of breastfeeding in Ghana. Although there is much evidence of government political will, a key gap identified was the inability to quantify government resources allocated to breastfeeding which was partly linked to the government budgeting system which does not directly earmark funds to breastfeeding. This is because breastfeeding is mainstreamed into other headline programs such as nutrition, child health, and growth promotion.

Gaps were also identified regarding the training & program delivery gear. These gaps were related to sub-optimal coordination of staff training initiatives, inconsistencies in training curricula and tools, unavailability of records on staff training, and the lack of certified lactation experts and trainers. The committee also highlighted gaps in the enforcement, monitoring, and evaluation of breastfeeding programs. The lack of routine monitoring of breastfeeding counseling was noted for attention. While these gaps were not necessarily new, the BBF provided an opportunity to identify them in a systematic and prioritized fashion in the context of a multi-sectoral process.

BBF was feasible in Ghana, yet it encountered multiple practical difficulties. Most prominent was the difficulty of accessing data needed to score the benchmarks. The barriers to accessing the needed data was due to three main reasons. First, the needed data was not immediately available from existing data bases. Second, key informants who could provide the needed information were not easily reachable to be interviewed. Third, many country committee members were extremely busy officials, and this affected the frequency of team meetings to identify and formulate data needs strategy early enough in the process.

Another key challenge was miscommunication and misperception between the BBF coordination team and the country committee gear team leaders. The Ghana BBF coordination team expected the gear teams to obtain data on their own and to request assistance from the Ghana BBF coordination team if they needed support with data collection. On the other hand, the gear teams expected that all the data required to complete their task would be supplied by the Ghana BBF coordination team. This misperception and miscommunication caused significant delays for the gear teams’ work, especially between the first and second committee meeting. Because these communication challenges caused delays in BBF implementation in Ghana, any future BBF assessment should clearly spell out the expectations of team members and to place the burden of data collection on the coordination team. This approach will avoid data collection challenges, minimize delays and ensure more efficient gear team action.

Despite its acceptance, some committee members indicated that some BBF benchmarks should be either excluded or revised because these benchmarks were not relevant to the Ghanaian context. Secondly, some benchmarks were thought to be useful only on an academic level and did not serve a practical purpose. Further, committee members reported underestimating the effort and time needed to implement the BBF. While these challenges were noted by the investigator as important feedback for improving the BBF process, committee members were encouraged to complete scoring of the benchmarks as outlined in the manual to accomplish the pretest as designed.

Several lessons have been learned from the implementation of BBF in Ghana. First is the need to ensure committee members are well informed about their roles to ensure they do not underestimate what is expected of them. Feedback from committee members indicated almost unanimous sentiment that BBF placed significant demand on their time and that they had underestimated the time requirements to complete the assigned team and committee activities. A second lesson is to be strategic in selecting committee members to ensure increased sectoral diversity of committee members and to include mid-level staff of the respective participating institutions who report to but may not be as busy as higher-level officials. Thirdly, there is need to foster awareness of BBF among high level decision makers to make it easier for the uptake of the recommendations generated from the process. Strategic engagement of high level decision makers in the beginning of BBF implementation can facilitate this better.

Finally, it is important to note that the BBF process does not directly assess specific infant feeding options, such as infant formula. However, it included benchmarks that assess how friendly the environment is to infant formula marketing, or otherwise. This assessment is expressed in two benchmarks; one in the legislation and policy gear, and the other in the research and evaluation gear.

## Conclusions

BBF was a feasible, multi-sectoral collaborative process that yielded a systematic measure of the Ghana breastfeeding enabling environment, identified gaps, and recommended actions for relevant sectors. BBF was well received and ongoing research is assessing the degree of adoption and implementation of the recommendations for scaling-up coverage for breastfeeding protection, promotion and support in Ghana.

## Additional files

Additional file 1: **Appendix S1.** Benchmark scores. This table presents the benchmark scores as well as gear total scores generated by the Becoming Breastfeeding Friendly Country committee in Ghana. (DOCX 18 kb)

Additional file 2: **Appendix S2.** Infographic. This document is an infographic which was developed and shared with key stakeholders in breastfeeding in Ghana who were invited to participate in the fifth committee meeting of the Becoming Breastfeeding Friendly Committee. (PDF 860 kb)

Additional file 3 **Appendix S3.** Policy Brief. This document is a policy brief developed and shared with high level decision makers across government and non-government stakeholders who participated in a high level decision makers consultation aimed at increasing awareness and uptake of the findings of the Becoming Breastfeeding Friendly Toolbox in Ghana. (PDF 1017 kb)
