# Supplementary material for: Pilot testing of the Becoming Breastfeeding Friendly toolbox in Ghana
Source: Int Breastfeed J. 2018 Jul 11;13:30. doi: 10.1186/s13006-018-0172-y (PMC6042403; doi:10.1186/s13006-018-0172-y)
Supplement: Supplementary file 1 — Appendix S1. Benchmark scores. This table presents the benchmark scores as well as gear total scores generated by the Becoming Breastfeeding Friendly Country committee in Ghana. (DOCX 18 kb) [file 13006_2018_172_MOESM1_ESM.docx]

Appendix 1: Benchmark scores by consensus by becoming Breastfeeding Friendly committee in Ghana, 2016

| Gear | Benchmark | Benchmark Score | Gear Total Score |
| --- | --- | --- | --- |
| Advocacy | AG1. There have been major events that have drawn media attention to breastfeeding issues.^1^ | 2 | 9 |
|  | AG2. There are high-level advocates (i.e. 'champions') or influential individuals who have taken on breastfeeding as a cause that they are promoting.^2^ | 3 |  |
|  | AG3. There is a national advocacy strategy based on sound formative research. | 3 |  |
|  | AG4. A national cohesive network(s) of advocates exists to increase political and financial commitments to breastfeeding. | 1 |  |
| Political Will | PWG1. High level political officials have publicly expressed their commitment to breastfeeding action.^3^ | 2 | 7 |
|  | PWG2. Government initiatives have been implemented to create an enabling environment that promotes breastfeeding.^4^ | 2 |  |
|  | PWG3. An individual within the government has been especially influential in promoting, developing, or designing breastfeeding policy.^5^ | 3 |  |
| Legislation and Policy | LPG1. A national policy on breastfeeding has been officially adopted/approved by the government.^6^ | 3 | 23 |
|  | LPG2. There is a national breastfeeding plan of action. | 3 |  |
|  | LPG3. The national BFHI/Ten Steps criteria has been adopted and incorporated within the healthcare system strategies/policy.^7^ | 3 |  |
|  | LPG4. The International Code of Marketing of Breast Milk Substitutes has been adopted in legislation.^8^ | 3 |  |
|  | LPG5. The National Code of Marketing of Breast Milk Substitutes has been enforced.^9^ | 1 |  |
|  | LPG6. The International Labour Organization Maternity Protection Convention has been ratified. | 2 |  |
|  | LPG7. There is paid maternity leave legislation for women.^10^ | 2 |  |
|  | LPG8. There is legislation that protects and supports breastfeeding or expressing breaks for lactating women at work.^11^ | 3 |  |
|  | LPG9. There is legislation for supporting worksite accommodations for breastfeeding women. | 0 |  |
|  | LPG10. There is legislation providing employment protection and prohibiting employment discrimination against pregnant and breastfeeding women.^12^ | 3 |  |
| Funding and Resources | FRG1: There is a national budget line(s) for Breastfeeding protection, promotion, and support activities. | 0 | 5 |
|  | FRG2: The budget is adequate for the Breastfeeding protection, promotion, & support activities. | 0 |  |
|  | FRG3: There is at least one fully funded government position to primarily work on BF protection, promotion and support at the national level. | 3 |  |
|  | FRG4: There is a formal mechanism through which maternity entitlements are funded using public sector funds. | 2 |  |
| Training and Program Delivery | TPDG1: Health provider schools & pre-service educ. programs for health care professionals that will care for mothers, infants & young children indicates curricula covers BF essential topics. | 1 | 33 |
|  | TPDG2: Facility-based health care professionals who care for mothers, infants & young children are trained on the essential BF topics as well as their responsibilities under the Code implementation. | 2 |  |
|  | TPDG3: Facility-based health care professionals who care for mothers, infants & young children receive hands-on training in essential topics for counseling & support skills for BF. | 2 |  |
|  | TPDG4: Community-based health care professionals who care for mothers, infants & young children are trained on the essential BF topics as well as their responsibilities under the Code implementation. | 2 |  |
|  | TPDG5: Community-based health care professionals who care for mothers, infants & young children receive hands-on training in essential topics for counseling & support skills for BF. | 2 |  |
|  | TPDG6: Community health workers & volunteers that work with mothers, infants & young children are trained on the essential BF topics as well as their responsibilities under the Code implementation. | 1 |  |
|  | TPDG7: Community health workers & volunteers that work with mothers, infants & young children receive hands-on training in essential topics for counseling & support skills for BF. | 1 |  |
|  | TPDG8: There exists national/subnational master trainers in BF (i.e. BF specialists or lactation consultants) who give support & training to facility-based & community-based health care professionals as well as community health workers. | 2 |  |
|  | TPDG9: BF training programs that are delivered by different entities through different modalities are coordinated (e.g.: face-to-face; on-line learning). | 2 |  |
|  | TPDG10: BF information & skills are integrated into related training programs (e.g., maternal & child health, IMCI). | 3 |  |
|  | TPDG11: Standards & guidelines for BF promotion & support have been developed & disseminated to all facilities & personnel providing maternity & newborn care. | 2 |  |
|  | TPDG12: Assessment systems are in place for designating BFHI/Ten Steps facilities. | 3 |  |
|  | TPDG13: Reassessment systems are in place that reevaluate Baby-Friendly/Ten Steps hospitals or maternity services to determine if they continue to adhere to the Baby Friendly/Ten Steps criteria. | 2 |  |
|  | TPDG14: More than 66.7% of hospitals & maternity facilities offering maternity services have been designated or reassessed as “Baby Friendly” in the last 5 years. | 2 |  |
|  | TPDG15: Health facility-based community outreach & support activities related to BF are being implemented. | 3 |  |
|  | TPDG16: Community-based BF outreach & support activities have national coverage. | 2 |  |
|  | TPDG17: There are trained & certified lactation management specialists available to provide supportive supervision for BF program delivery. | 1 |  |
| Promotion | PG1: There is a national BF promotion strategy that is grounded in the country’s context. | 3 | 6 |
|  | PG2: The national BF promotion strategy is implemented. | 1 |  |
|  | PG3: Government or civic organizations have raised awareness about BF. | 2 |  |
| Research and Evaluation | REG1: Indicators of key BF practices are routinely included in periodic national surveys. | 3 | 13 |
|  | REG2: Key BF practices are monitored in routine health information systems. | 2 |  |
|  | REG3: Data on key BF practices are available at national & sub-national, including local/municipal, levels. | 2 |  |
|  | REG4: Data on key BF practices are representative of key vulnerable groups. | 0 |  |
|  | REG5: Indicators of key BF practices are placed in the public domain on a regular basis. | 2 |  |
|  | REG6: A monitoring system is in place to track implementation of the Code. | 1 |  |
|  | REG7: A monitoring system is in place to track enforcement of maternity protection legislation. | 0 |  |
|  | REG8: A monitoring system is in place to track provision of lactation counseling/management & support. | 1 |  |
|  | REG9: A monitoring system is in place to track implementation of BFHI/Ten Steps. | 2 |  |
|  | REG10: A monitoring system is in place to track behavior change communication activities. | 0 |  |
| Coordination Goals & Monitoring | CGMG1: There is a National BF Committee/ IYCF Committee. | 2 | 8 |
|  | CGMG2: National BF Committee/IYCF committee work plan is reviewed & monitored regularly. | 3 |  |
|  | CGMG3: Data/information related to BF program progress are used for decision-making & advocacy. | 3 |  |
| Adapted from Fox et al. (2015)  2 Adapted from Fox et al. (2015)  3 Adapted from Fox et al. (2015)  4 Adapted from Fox et al. (2015)  5 Adapted from Fox et al. (2015)  6 Adapted from the WBTi (2014).  7 Adapted from WHO’s Infant and Young Child Feeding assessment tool (2003)  8 Adapted from the WBTi (2014).  9 Adapted from the WBTi (2014).  9 Adapted from the WBTi (2014).  ^0^ Adapted from the WBTi (2014).  ^1^ Adapted from the WBTi (2014). | | | |
