# Supplementary material for: Pilot testing of the Becoming Breastfeeding Friendly toolbox in Ghana
Source: Int Breastfeed J. 2018 Jul 11;13:30. doi: 10.1186/s13006-018-0172-y (PMC6042403; doi:10.1186/s13006-018-0172-y)
Supplement: Supplementary file 2 — Appendix S2. Infographic. This document is an infographic which was developed and shared with key stakeholders in breastfeeding in Ghana who were invited to participate in the fifth committee meeting of the Becoming Breastfeeding Friendly Committee. (PDF 860 kb) [file 13006_2018_172_MOESM2_ESM.pdf]

# bbf becoming breastfeeding friendly

A GUIDE TO GLOBAL SCALE-UP

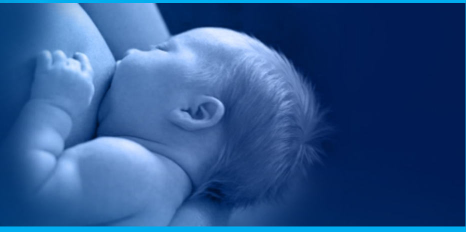

## BREASTFEEDING IN GHANA

Breastfeeding SAVES LIVES by reducing the risk of disease and death of mothers and infants during the first few months of life and into childhood and adulthood.

World Health Organization recommends initiation of breastfeeding within the first hour after birth and exclusive breastfeeding (i.e. breastmilk only) for the first 6 months of a child's life.

The **BBF** guides countries with assessing and scaling up their breastfeeding protection, promotion, and support programmes nationally.

**56%** of Ghanaian infants are introduced to breast milk within1 hour of birth

**52%** of infants under 6 months are fed exclusively with breastmilk.

**BBF Objective:** Understand why breastfeeding rates are suboptimal and recommend how to improve them through large scal e programs.

**BBF:**
Provides low, middle, and high income countries with an evidence-based index comprised of 54 benchmarks and 87 case studies, identified from a multitude of countries.

Based on the Breastfeeding Gear Model that stipulates eight "gears" – Advocacy, Political Will, Legislation & Policies, Funding & Resources, Training & Program Delivery, Promotion, Research & Evaluation, and Coordination, Goals & Monitoring – that must be at work and in harmony for large-scale improvement in a country's national breastfeeding program

BBF not only assesses and measures, but also supports countries to scale-up BF programmes through step-by-step criteria.

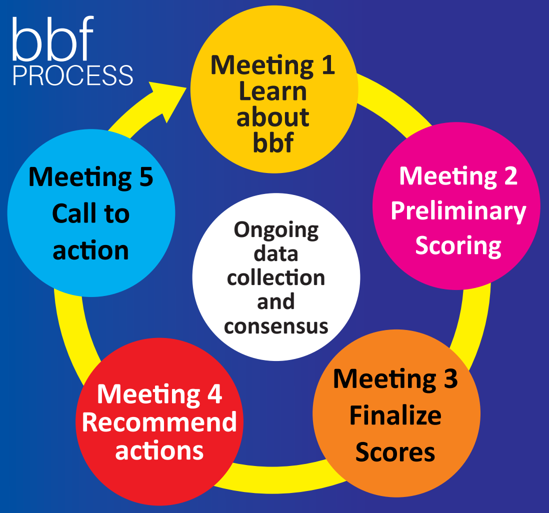


## SCORE LEGEND

| Gear Total Score | Interpretation |
|---|---|
| 0 | [illegible]ear not present |
| 0.1 to 1.0 | Weak Gear Strength |
| 1.1 to 2.0 | Moderate Gear Strength |
| 2.1 to 3.0 | Strong Gear Strength |

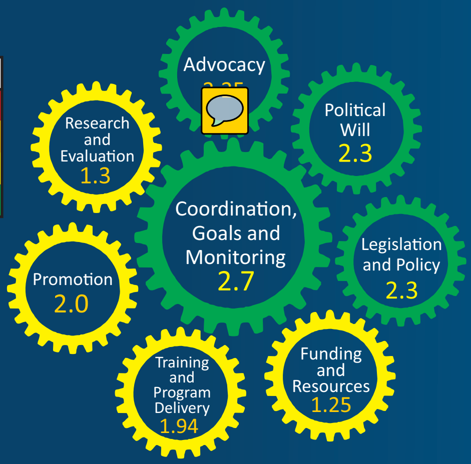


## KEY RECOMMENDATIONS

1. Strengthen breastfeeding advocacy efforts and empower potential champions.

2. Strengthen BFHI: Support ongoing decentralization of BFHI implementation process.

3. Strengthen training ~~and~~ program delivery: Ensure content of all training programs curricula maintains adequate program standards for breastfeeding

4.Strengthen breastfeeding monitoring ~~and~~ reporting: maintain a surveillance of trained personnel ~~and~~ training activities

5.Strengthen maternity protection: adopt ILO Maternity Protection Convention 2000, (No. 183) ~~and~~ increase paid maternity leave duration to at least 14 weeks.

6.Strengthen ~~the~~ WHO Code by ensuring strong monitoring at all levels.

7.Enhance behaviour change communication activities by engaging multiple provider constituencies including retired staff ~~and~~ community based volunteers

*Pérez-Escamilla et al. Adv Nutr (2012)*

UNIVRESITY OF GHANA SCHOOL OF PUBLIC HEALTH

FAMILY LARSSON ROSENQUIST FOUNDATION dedicated to human milk

YALE
