# Supplementary material for: Pilot testing of the Becoming Breastfeeding Friendly toolbox in Ghana
Source: Int Breastfeed J. 2018 Jul 11;13:30. doi: 10.1186/s13006-018-0172-y (PMC6042403; doi:10.1186/s13006-018-0172-y)
Supplement: Supplementary file 3 — Appendix S3. Policy Brief. This document is a policy brief developed and shared with high level decision makers across government and non-government stakeholders who participated in a high level decision makers consultation aimed at increasing awareness and uptake of the findings of the Becoming Breastfeeding Friendly Toolbox in Ghana. (PDF 1017 kb) [file 13006_2018_172_MOESM3_ESM.pdf]

Becoming Breastfeeding Friendly

Recommended Actions to Scale Up Breastfeeding Impact in Ghana

Policy Brief

Janauary 2017

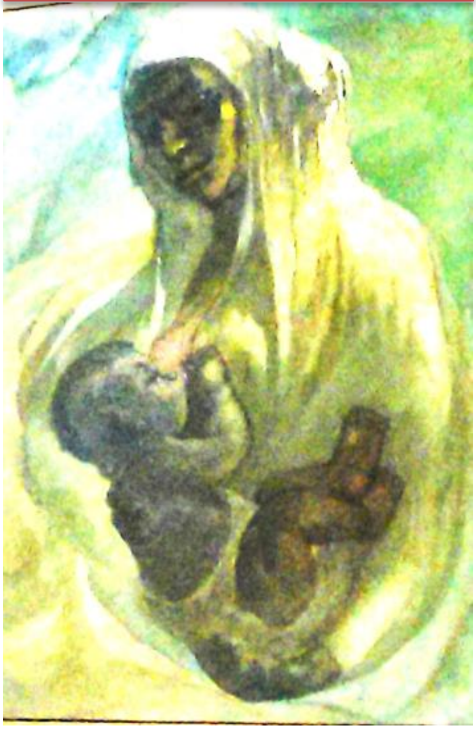

Key Messages:

- Timely initiation and exclusive breastfeeding duration in Ghana falls below national targets.
- Key program areas needing improvement include advocacy targeting decision makers, and awareness to general public on breastfeeding, maternity protection, and strong capacity for service delivery
- Multi-sectoral commitments are needed for scaling up recommended actions arising from the becoming Breastfeeding Friendly Process in Ghana.

Background

Breast milk provides all of the essential nutrients, minerals, and vitamins an infant requires for growth in their first months of life. It also contains antibodies that help combat disease and build the child's immunity at the most vulnerable part of his or her life. During the first six months, an exclusively breastfed child (given only breast milk) is 14 times less likely to die than a non-breastfed child.

Early initiation and exclusive breastfeeding of young children are essential components of Ghana's child health Strategy. However, only 56% of Ghanaian infants are put to the breast within one hour of delivery, as recommended. Further, only 52% of infants younger than 6 months are exclusively breastfed compared to 63% in 2008. Indeed, the median duration of exclusive breastfeeding has been progressively declining over the past decade and is now at only 2.5 months.

To contribute to addressing these adverse outcomes, the Becoming Breastfeeding Friendly (BBF) process used the ***Breastfeeding Gear Model (BGM)***[1] to assess country readiness to scale up various components of breastfeeding services in Ghana (programs, initiatives, policy environment, etc). The BBF will also help countries use evidence to scale up breastfeeding support services. The BGM uses available contextual evidence to score country breastfeeding performance based on 8 'Gears':

- Advocacy
- Political Will
- Legislation and Policy
- Funding and Resources
- Training and Program Delivery
- Promotion
- Research and Evaluation
- Coordination, Goals, and Monitoring

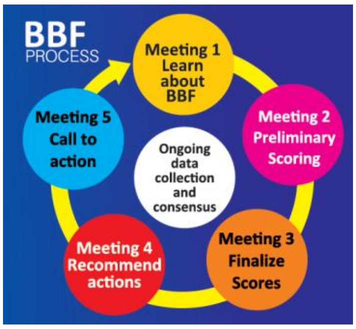

Who is this policy brief for?

MOH/GHS, UNICEF, WHO, ILO, GINAN

Why was this policy brief prepared?

The Becoming Breastfeeding Friendly Project identified gaps national breastfeeding programming. The Following recommended actions have been prepared for decision makers to contribute to addressing the gaps

This evidence-based policy brief includes:

- Background of Ghana's breastfeeding situation
- Process and findings of the BBF process
- Recommended key actions to scale up breastfeeding

[1] Perez-Escamilla et al ***Advances in Nutrition*** (2012)

becoming breastfeeding friendly
A GUIDE TO GLOBAL SCALE-UP

UNIVERSITY OF GHANA
SCHOOL OF PUBLIC HEALTH

In consultation with the Ghana Health Service, a 12-member committee with membership from government, United Nations agencies, civil societies, bilateral donor organizations, and academia was established to implement the process, in collaboration with the Ghana Health Service and under the leadership of the University of Ghana. The committee held four meetings between June and December 2016. Using a rigorous scoring process, the committee scored the national breastfeeding program performance, by consensus, on the 8 gears using best available evidence. The figure below is color-coded and shows the outcome of the readiness assessment for each of the 8 gears. Overall, the country score was 1.99 out of a maximum of 3.0. This means the country has a moderate strength environment for scaling up breastfeeding impact.

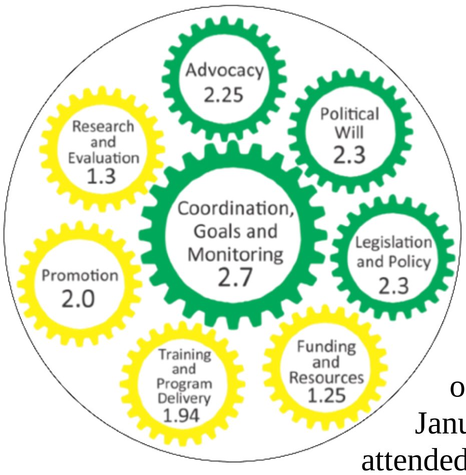

SCORE LEGEND

Gear Total Score	Interpretation
0	Gear not present
0.1 to 1.0	Weak Gear Strength
1.1 to 2.0	Moderate Gear Strength
2.1 to 3.0	Strong Gear Strength

Following the assessment, the committee also proposed recommendations for action based on the BBF findings. Subsequently in January 2017, a call to action meeting, attended by a cross-section of stakeholders, enabled sharing, discussion, and prioritization of the key recommendations. Below are a list of the key recommendations and priority actions for the consideration of decision makers and breastfeeding champions.

Key prioritized Recommendations

1. Enlist/Engage Breastfeeding Champions

Key recommendation:

Strengthen advocacy by enlisting more breastfeeding champions (high level and visible individuals); engage with existing champions and build their advocacy capacity:

Proposed actions:

- Develop advocacy materials to actively engage Ghana Journalists Association and Editors Forum
- Actively promote breastfeeding through existing scientific meetings of the professional associations/societies

Financial and social costs:

-

2. Strengthen Maternity Protection

Key recommendation:

Ratify and adopt provisions of ILO Maternity Protection Convention, 2000, No. 183

Proposed actions:

- Facilitate adoption of at least 14 weeks maternity leave
- Facilitate amendment of LI 1667 (Breastfeeding Promotion Law) to incorporate relevant World Health Assembly resolutions
- Strengthen implementation and monitoring of the Code of marketing of breast milk substitutes.
- Support incorporation of maternity protection as part of collective bargaining agreements with employers

Financial and social costs:

- Will cost $94/live birth per year, for six months paid leave
- Monitoring of the Code will cost $0.6/live birth per year

3. Capacity strengthening

Key recommendation:

Harmonize, strengthen, and monitor pre-service and in-service breastfeeding training of health staff and volunteers providing breastfeeding services

Proposed actions:

- Harmonize all training curricula; eliminate contradictions, strengthen hands-on component of training
- Establish decentralized harmonized database of in-service training and trainers
- Establish certification for lactation management specialists/consultants

Financial and social costs:

- Will cost $2/live birth to train health workers per year
- Training of community volunteers will cost $10/live birth

4. Awareness creation

Key recommendation:

Scale up dissemination of accurate information on breastfeeding practice using multiple channels of communication at all levels

Proposed actions:

- Increase budget for implementation of social behaviour change communication via multiple media
- Sustain breastfeeding promotion all year round in partnership with media
- Engage retired health staff to support breastfeeding promotion

Financial and social costs:

- Media advertising will cost $5/live birth per year

Annex: Full list of recommendations

Advocacy Gear:

- Implement training (e.g. seminars) for media personnel across the country on breastfeeding issues to build their knowledge and capacity to advocate for breastfeeding interventions through the Ghana Journalist Association and the Editors Forum.
- Enlist new/Engage existing champions and provide materials on breastfeeding for them to use for advocacy
- Actively promote breastfeeding through existing scientific meetings of the professional associations/societies e.g. medical association, nutrition association, etc.
- Actively engage persons identified as breastfeeding champions and also professional societies (including GINAN) and build their capacity for effective advocacy; motivate them with system of recognition, e.g. as part of annual reviews
- Revise and update breastfeeding-relevant policies and strategies, and scale up implementation of such policies country-wide

Political Will Gear:

- Identify additional forums for sensitizing high level public officials on breastfeeding
- Produce and disseminate advocacy tools on breastfeeding; e.g. policy briefs for parliamentarians
- Ensure scale-up of Baby Friendly Hospitals initiative decentralization process
- Intensify advocacy for adoption of the maternity protection convention (ILO No 183) at high levels

Legislation & Policy Gear:

- Provide guidelines for setting realistic targets for breastfeeding at national level
- Ensure the 'Ten Steps to successful breastfeeding' are explicitly indicated in key documents on newborn and child health
- Revise LI 1667 (breastfeeding promotion Law) to allow more stringent penalties for Code violations and also incorporate recent World Health Assembly resolutions
- Strengthen implementation and dissemination of the Code of marketing of breast milk substitutes.
- Advocate for ratification of the ILO Maternity Protection Convention, 2000, No. 183.
- Facilitate adoption of at least 14 weeks maternity leave.
- Facilitate amendment of LI 1667 (breastfeeding promotion Law) to require onsite accommodations for breastfeeding in government and private work places
- Establish monitoring system regarding employment protection and discrimination relating to breastfeeding and maternity; will require gathering data on violations, particularly in the private sector.
- Maternity protection at workplace should be incorporated into trade union collective bargaining agreements.
- Strengthen enforcement of code of marketing of breast milk substitutes

Funding & Resources Gear:

- Review budget line indicators for reporting and tracking breastfeeding
- Breastfeeding should be adequately budgeted for in integrated annual work plans
- Analyze and report breastfeeding data in district-level health information management system (during annual reviews) for program improvement

Training & Program Delivery Gear:

Curricula

- Champion and implement updated breastfeeding training curricular of Nurses and Midwives Council in pre-service training institutions.
- Harmonize training curricula used by government health service agencies and also non-government partners; eliminate contradictions, ensure it contains key competencies (knowledge and skills aspects) necessary for optimal practice.
- Update Community Infant and Young Child Feeding (IYCF) training package to include health worker responsibilities under the Code
- Strengthen practical component of training using 'qualified trainers'

M & E

- Establish decentralized harmonized database of in-service training (+ trainers) to ensure adequate information on existing capacity and gaps.
- Assess content of breastfeeding training programs to identify gaps.
- Establish capacity need for breastfeeding
- Establish/strengthen monitoring system for monitoring BFHI re-assessment-together with an alert system that kicks in prior to the time for reassessment; strengthen decentralization of BFHI reassessment

Job aids and resources

- Ensure timely dissemination of training documentation to all stakeholders; keep records of institutions which have received the documents.

Training programs

- Scale up community health workers and volunteers training in breastfeeding
- Target training and service delivery resources to districts which are resource constrained
- Operationalize process to certify lactation management specialists/consultants

Promotion Gear:

- Scale up dissemination/implementation of existing policies and programs on breastfeeding
- Increased budget for implementation of social behaviour change communication campaigns
- Engage retired health staff (as complement to staff in service) and also volunteers to support breastfeeding promotion
- Report breastfeeding programming activities (including promotion activities) at decentralized levels
- Develop program for breastfeeding promotion all year round in partnership with media
- Use maternity promotion as a tool for breastfeeding promotion
- Awareness creation among workers concerning existing maternity protection should be implemented

Research & Evaluation Gear:

- Operationalize annual breastfeeding surveillance system
- Identify groups with elevated vulnerability regarding successful breastfeeding and establish breastfeeding protection, promotion and support criteria for these vulnerable groups
- Ensure wide dissemination of national survey data on breastfeeding
- Decentralize monitoring of Code implementation
- Develop simplified template for collation of reports on lactation counselling/support to be reported at regional level
- Create access to BFHI/Ten Steps implementation tracking data on Ghana Health Service (GHS) website
- Include behaviour change communication tracking data in district-level health information management system

Coordination, Goals, & Monitoring Gear:

- Ensure regular scheduled meetings of BFHI/IYCF committees are implemented; limit adhoc meetings
- Develop a workplan for action
